# Tribute to Bernard Cohen - Whose Pioneering Work Made the Vestibular Implant Possible

**DOI:** 10.3389/fneur.2020.00452

**Published:** 2020-05-27

**Authors:** Jean-Philippe Guyot, Nils Guinand, Angelica Perez Fornos

**Affiliations:** Division of ENT and Head-and-Neck Surgery, Geneva University Hospitals, Geneva, Switzerland

**Keywords:** vestibular system, vestibular loss, vestibulopathy, electrical stimulation, vestibular implant, cochlear implant, neuroprosthesis, nystagmus

It is estimated that ~1.8 million adults suffer from a severe or total bilateral vestibular deficit worldwide (1). Despite the dramatic consequences of the disease on the physical, emotional, and social functioning (2) in adults as well as its negative effects on the development of children born without vestibular function (3), there is no effective treatment for these patients (4). It was not until the mid-1990s that the idea of developing a neuroprosthesis that provides position and motion information to the brain using a concept comparable to the cochlear implant was born. Undoubtedly, this idea was based on the pioneering work of Bernard Cohen and his colleague Jun-Ichi Suzuki who, in the sixties, obtained and precisely described the reflex responses obtained by electric stimulation of the ampullary nerves in rabbits, pigeons, cats, and monkeys (5–8). Three decades later, Merfeld and Gong demonstrated that a rotation signal could be delivered to the nervous system using a piezoelectric gyroscope modulating the frequency of electrical signals according to the direction and the speed of head movements in guinea pigs (9, 10). It was time to move on to experimentation in humans.

It then seemed reasonable to us to see whether it was possible to duplicate the experiences of Cohen and Suzuki (5–8) in humans. In other words, we wanted to explore the possibility of generating vestibular reflexes upon electrical stimulation of the branches of the vestibular nerve, while at the same time limiting possible risks of hearing loss caused by the introduction of electrodes in the inner ear. Surgical approaches to the posterior and lateral ampullary nerves were developed (11) and, in 2004, the first electrical stimulation trials were performed in local anesthesia in patients undergoing surgery for cochlear implantation or suffering from Menière's disease eligible for a surgical labyrinthectomy.

These experiments showed that it was possible to access the branches of the vestibular nerve surgically without opening the labyrinth and that, not surprisingly, and in agreement with the pioneering works of Cohen and Suzuki, the nystagmic responses were aligned with the plane of the stimulated canal (12–14). Since 2007, this has led to several implantations of our vestibular implant prototypes in humans, with the demonstration of partial restoration of the vestibular function (11). Other groups in Baltimore and Washington followed with promising outcomes (15, 16).

We owe a lot to Bernard Cohen for his contribution in the field of vestibular physiology which opened the door to the development of a vestibular implant. This raises high hopes to improve the quality of life of patients suffering from a bilateral deficit. As for us, we were thrilled to present these first results at the meeting of the Association Research in Otolaryngology in Baltimore in 2011: Bernard Cohen was part of the audience. Thank you for the inspiration and encouragement, Sir !

We were sad to learn that he passed away in Mont Sinai Hospital on November 27 2019 the same hospital where he had initiated the original studies almost six decades ago.

## Author Contributions

J-PG, NG, and AP wrote and approved the manuscript.

### Conflict of Interest

The authors declare received research and travel grants from MED-EL Elektromedizinische Geräte GMBH (Innsbruck, Austria). The remaining authors declare that the research was conducted in the absence of any commercial or financial relationships that could be construed as a potential conflict of interest.
